# anndataR improves interoperability between R and Python in single-cell transcriptomics

**DOI:** 10.1093/bioinformatics/btag288

**Published:** 2026-05-09

**Authors:** Louise Deconinck, Luke Zappia, Robrecht Cannoodt, Martin Morgan, Isaac Virshup, Chananchida Sang-Aram, Danila Bredikhin, Brian Schilder, Ruth Seurinck, Yvan Saeys, scverse core, Can Ergen, Danila Bredikhin, Emma Dann, Giovanni Palla, Gregor Sturm, Ilan Gold, Isaac Virshup, Jennifer A Foltz, Luca Marconato, Lukas Heumos, Mikaela Koutrouli, Pau Badia-i-Mompel, Philipp Angerer, Roshan Sharma, Sara Jimenez, Severin Dicks, Tim Treis, Wouter-Michiel Vierdag

**Affiliations:** Data Mining and Modelling for Biomedicine, VIB Center for Inflammation Research, 9000 Ghent, Belgium; Department of Mathematics, Computer Science and Statistics, Ghent University, 9000 Ghent, Belgium; Data Intuitive, 9280 Lebbeke, Belgium; Data Mining and Modelling for Biomedicine, VIB Center for Inflammation Research, 9000 Ghent, Belgium; Department of Mathematics, Computer Science and Statistics, Ghent University, 9000 Ghent, Belgium; Data Intuitive, 9280 Lebbeke, Belgium; Edaro, 9070 Destelbergen, Belgium; Department of Biostatistics and Bioinformatics, Roswell Park Comprehensive Cancer Center, NY 14263, United States; Institute of Computational Biology, Helmholtz Center Munich, D-85764 Neuherberg, Germany; Chan Zuckerberg Initiative, Redwood City, CA 94083, United States; Data Mining and Modelling for Biomedicine, VIB Center for Inflammation Research, 9000 Ghent, Belgium; Department of Mathematics, Computer Science and Statistics, Ghent University, 9000 Ghent, Belgium; Department of Genetics, Stanford University, Stanford, CA 94305, United States; scverse; Department of Brain Sciences, Faculty of Medicine, Imperial College London, W12 0NN London, United Kingdom; Data Mining and Modelling for Biomedicine, VIB Center for Inflammation Research, 9000 Ghent, Belgium; Department of Mathematics, Computer Science and Statistics, Ghent University, 9000 Ghent, Belgium; Data Mining and Modelling for Biomedicine, VIB Center for Inflammation Research, 9000 Ghent, Belgium; Department of Mathematics, Computer Science and Statistics, Ghent University, 9000 Ghent, Belgium

## Abstract

**Summary:**

Many single-cell transcriptomics datasets are stored in the HDF5-backed AnnData (H5AD) file format, as popularized by the Python scverse ecosystem. However, accessing these datasets from R, allowing users to take advantage of the strengths of each language, can be difficult. anndataR facilitates this access by allowing users to natively read and write H5AD files in R, convert them to and from SingleCellExperiment or Seurat objects, or even work with the resulting R AnnData object directly. We perform rigorous testing to ensure compatibility between Python-written and R-written H5AD files, guaranteeing long-term interoperability between languages.

**Availability and implementation:**

anndataR’s source code is available on GitHub at scverse/anndataR under the MIT license. It is compatible with R version 4.5, has been archived at 10.5281/zenodo.18775712 and included within Bioconductor: 10.18129/B9.bioc.anndataR. Installation instructions and tutorials can be found in the online documentation at anndatar.scverse.org. Issues can be reported at the GitHub repository. Code to reproduce the analyses performed can be found on GitHub at LouiseDck/anndataR-paper, archived at 10.5281/zenodo.18792241.

## Graphical abstract

**Graphical Abstract:**
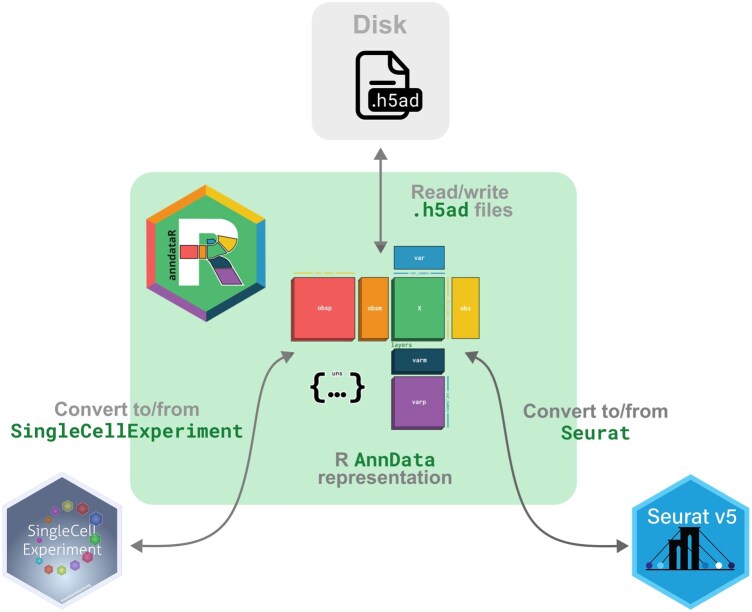

## 1 Introduction

In the single-cell transcriptomics field, three main analysis ecosystems exist: scverse (Virshup *et al.* 2023), Seurat (Satija *et al.* 2015), and Bioconductor (Amezquita *et al.* 2020), each defining its own in-memory data format. Two of these, the SingleCellExperiment object used by Bioconductor and the Seurat object, are implemented in R, while the scverse AnnData object (Virshup *et al.* 2024) is Python-based.

Each ecosystem has specific advantages for single-cell transcriptomics (scRNA-seq) data analysis, and best practices and benchmarks recommend combining tools from different ecosystems to obtain an optimal processing pipeline for scRNA-seq data (Heumos *et al.* 2023). For instance, Seurat easily accommodates multi-modal assays, while Bioconductor comes with easy access to extensive statistical tooling, and scverse leverages scalability and access to machine learning ecosystems. On top of that, small implementation differences in basic functionality (e.g. PCA) between the ecosystems may produce significantly different results (Rich *et al.* 2026). As a result, users may need to switch between data formats or even programming languages to perform different analysis steps or to replicate existing analyses. Unfortunately, this is not straightforward, and a number of issues arise when trying to convert data between formats. These issues stem from (i) structural differences between the data formats and (ii) the different programming languages.

### 1.1 Differences in structure

All three data formats were developed to work with single-cell transcriptomics data, but they take different approaches and structure their objects differently. An overview of the structure of these objects can be found in Table 1.

**Table 1:** Overview of the three different data formats and the slots where specific information is stored.

Stored information	AnnData	SCE	Seurat v5
Expression matrices	X, layers	assays	layers
Unfiltered expression matrices (different features)	raw	altExp	layers
Paired measurements (spike in, CITE-seq, …)		altExp	assays
Multi-omics data (different cells)			assays
Cell-level metadata (cell type, …)	obs	colData	cell level metadata
Feature-level metadata (number of cells, …)	var	rowData	assay level metadata
Multidimensional cell-level metadata (dimensionality reductions)	obsm	reducedDims	reductions
Multidimensional feature-level metadata (loadings of dimensionality reductions)	varm	reducedDims	reductions
Cell by cell similarity matrices	obsp	colPairs	graphs
Feature by feature similarity matrices	varp	rowPairs	
Other unstructured metadata	uns	metadata	misc

For example, the AnnData object contains a varm slot that stores multidimensional variable annotations (such as PCA loadings), but SingleCellExperiment and Seurat instead store this information as metadata of the cell reduction objects. The Seurat object also has no corresponding slot for the AnnData varp slot, which is used for pairwise variable-level annotations. Seurat and SingleCellExperiment can also store multimodal data, which is not possible in AnnData. Additionally, the frameworks differ in matrix orientation conventions. AnnData uses an observation-by-feature layout, with observations (cells) in rows and features (genes) in columns. Conversely, SingleCellExperiment and Seurat implement these matrices with cells as columns and genes as rows. Because of these structural differences, in-depth knowledge of each object and the related storage conventions is required to correctly convert between formats, which presents a barrier to users moving between ecosystems.

### 1.2 Different programming languages

The different programming languages also complicate interoperability. Seurat and Bioconductor are written in R, while scverse is written in Python. As mentioned earlier, tools in both programming languages are required to optimally analyze a single dataset. To address this, two other core data structures within the scverse ecosystem have some cross-language interoperability: MuData (Bredikhin *et al.* 2022), designed for multimodal data, provides R and Julia implementations, and SpatialData (Marconato *et al.* 2025) maintains active collaboration with R developers (Marconato *et al.* 2024). For single-cell transcriptomics analysis, there are many software packages that facilitate the conversion between different objects (Table 2). They use a combination of approaches to transfer data between programming languages: they either use a so-called foreign function interface (FFI) to transfer information between languages during an interactive session, or they write and read the data to and from disk.

**Table 2:** Overview of packages aiming to provide some form of interoperability between AnnData, SingleCellExperiment and Seurat objects.

Tool	Prog. language	FFI	File format	Conversion
dynverse/anndata (Cannoodt 2025)	R	√		
anndata2ri (Angerer *et al.* 2020)	Python	√		AnnData↔SCE
Loom	R, Python		Loom (h5)	
scDIOR (Feng *et al.* 2022)	R, Python		scDIOR (h5)	AnnData↔SCE AnnData↔Seurat
BiocPy with rds2py	Python			
alabaster (Lun 2024) dolomite	R, Python		HDF5, csv	
SeuratDisk	R		h5Seurat	Seurat↔AnnData
zellkonverter (Zappia and Lun 2025)	R	√		AnnData↔SCE
sceasy (Cakir *et al.* 2020)	R	√		Converts between AnnData, SCE, Seurat and Loom
schard	R			Converts between AnnData, SCE and Seurat^a^
anndataR (this paper)	R			Converts between AnnData, SCE and Seurat
SCUBA (Showers *et al.* 2024)	R	√	provides API	
scKirby	R	√		Converts between AnnData, SCE, Seurat and Loom^b^

a Incomplete: only reads and converts X, obs, var, obsm.

b Combines conversion functionality from other packages.

FFIs like reticulate (https://CRAN.R-project.org/package=reticulate) or rpy2 (https://github.com/rpy2/rpy2) allow a program written in one language to call or use functions written in another language. This allows users to flexibly call foreign functions, transferring data from the native language to the foreign language as needed. However, such an approach faces several limitations. It is limited to built-in datatypes (such as vectors, lists, or integers); requires managing both a Python and R environment; and naive use of these interfaces can lead to excessive memory usage. Software packages that use FFIs to facilitate interaction with single-cell-specific data formats include anndata2ri (Angerer et al. 2020) and dynverse/anndata (Cannoodt 2025).

The second approach, working on an object that gets intermediately stored on disk, requires a standard on-disk format accessible from both languages. A shared standard, the loom, was introduced but was not fully taken up by the community. The AnnData HDF5-based (https://github.com/HDFGroup/hdf5) H5AD format has become the de facto standard for Python users and in some public repositories, while R objects are commonly saved as non-interoperable binary Rds files. To address this, packages such as rds2py (https://github.com/BiocPy/rds2py), alabaster (Lun 2024), and dolomite (https://github.com/ArtifactDB/dolomite-base) provide ways to read and write SingleCellExperiment objects in Python. SeuratDisk (https://github.com/mojaveazure/seurat-disk) provides a way to save Seurat objects as interoperable HDF5 files.

Other packages, including sceasy (Cakir *et al.* 2020) and zellkonverter (Zappia and Lun 2025), combine these approaches to make H5AD files usable in R. They allow reading and writing of H5AD files to and from Seurat or SingleCellExperiment objects but do so by using the reticulate software package, an FFI, to read the H5AD file using the Python anndata package before converting formats. This approach only partially alleviates the burden of working with these FFIs. R users (or package maintainers) still need to maintain a Python environment and be mindful of in-memory duplication of data, even when all their analysis is performed in R. The data type conversions from Python to R can also be more cumbersome than those needed when reading the HDF5 file from disk.

## 2 Results

To overcome these limitations, we introduce anndataR, a new package that allows native reading and writing of H5AD files in R without requiring a Python environment, provides an R AnnData object, and facilitates conversion to and from other data formats. anndataR was conceived and developed as a collaborative effort between members of both the scverse and Bioconductor communities, who together identified the gaps in the current interoperability climate.

anndataR is unique in its ability to read and write H5AD files natively in R and convert them to SingleCellExperiment or Seurat objects. By avoiding FFIs, anndataR avoids many of the associated challenges. The conversion functionality it offers bridges the gap between the ecosystems, with sensible defaults (see Figs 2 and 3, available as supplementary data at *Bioinformatics* online) and fine-grained control for advanced use. anndataR allows users direct access to an R AnnData representation, making it possible to interact with the object directly to solve conversion issues and to facilitate easy extraction of relevant parts of the data.

## 3 Software design

**Figure 1:**
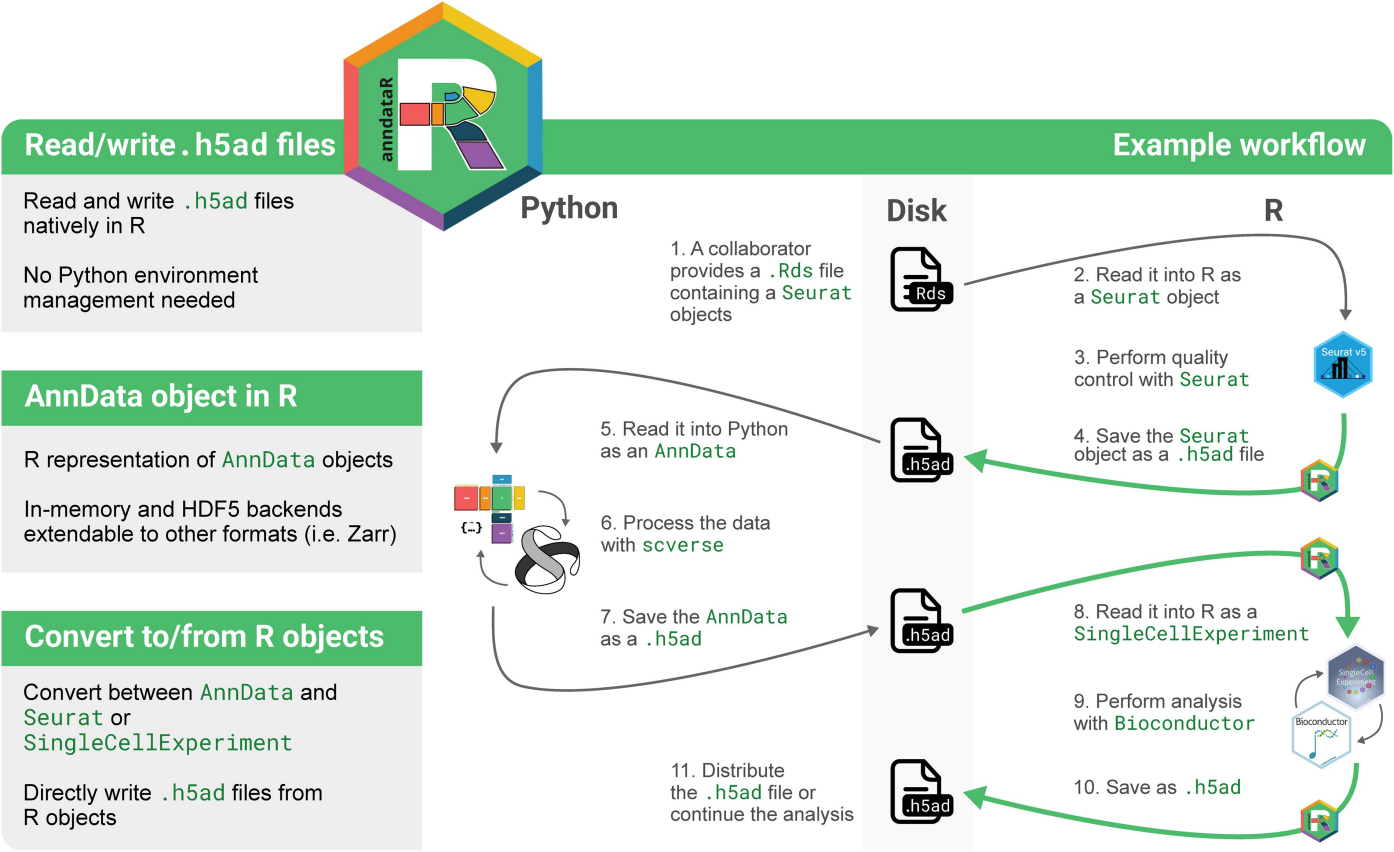
Feature overview and showcase of how to use anndataR in an analysis workflow.

Users primarily interact with anndataR by reading an existing H5AD file and specifying whether to return an AnnData object in R (either in memory or HDF5-backed) or to immediately perform a conversion to a SingleCellExperiment or Seurat object, as shown in Fig. 1, available as supplementary data at *Bioinformatics* online. The resulting R object can then be used as normal and can be saved to a new H5AD file on completion of the analysis. An example of how anndataR can be used in an analysis workflow is shown in Fig. 1.

To enable this workflow, we use rhdf5 (Fischer *et al.* 2025), an R interface to the HDF5 library that handles the low-level interactions with HDF5 files to natively read and write the H5AD format according to the AnnData on-disk specification. To take advantage of existing R analysis packages, users also need to be able to convert between AnnData objects and SingleCellExperiment or Seurat objects. By default, the anndataR conversion functions try to convert as much data as possible but also allow the experienced user to completely specify the details of the conversion by providing a mapping for each slot in the resulting object. Full details of the conversion can be found in the package documentation.

We are aware that small changes in any of these data formats can quickly lead to failures in reading, writing, or converting. To ensure the package functionality is robust against data format changes, we perform extensive testing. H5AD files written with anndataR are validated against the ones produced by the Python anndata package. This is necessary because there are differences in how Python and R handle data, such as row-major versus column-major matrices, and in how the Python and R HDF5 libraries translate data to H5 datatypes. We perform these checks using a number of round-trip tests for each AnnData slot, checking whether an R-written H5AD slot can be read in Python and vice versa. We also check for differences in the generated H5AD files using the h5diff utility. Additionally, we verify the conversions to and from SingleCellExperiment and Seurat by slot-by-slot verification of the objects. This approach to exhaustively testing functionality has been key for the development of anndataR, allowing us to document known limitations and incompatibilities. This has led to a comprehensive and robust tool (Table 1, available as supplementary data at *Bioinformatics* online) that is faster and more memory-efficient than comparable tools, particularly for larger datasets (Fig. 4, available as supplementary data at *Bioinformatics* online). Continuous benchmarking via Bencher (https://bencher.dev/perf/anndatar/plots) further ensures that performance does not regress over time.

Finally, in order to represent the complex AnnData structure, and replicate the Python interface, make use of inheritance, keep memory usage low, and use reference semantics, we chose to use the R6 object-oriented class system.

## 4 Conclusion

anndataR meaningfully adds to the interoperability landscape between R and Python for single-cell transcriptomics by allowing users to work natively with H5AD files in R and providing conversion functionality to and from SingleCellExperiment and Seurat objects. This conversion functionality not only provides reasonable defaults but also allows fine-grained user control. Additionally, the package functionality, such as compatibility between R-written and Python-written H5AD files, is rigorously tested.

Finally, the modular design of the software makes it easy to extend the functionality of the package, enabling future support for additional file formats (e.g. Zarr) and other modalities, such as scATAC-seq or CITE-seq (via MuData (Bredikhin *et al.* 2022)) and spatial data (via the SpatialData (Marconato *et al.* 2025) framework).

## Supplementary Material

btag288_Supplementary_Data

## Data Availability

No new experimental data were generated in the course of this study.
